# Predictors of exacerbation in myasthenia gravis after minimal symptom expression: a bicenter cohort study

**DOI:** 10.3389/fimmu.2026.1786218

**Published:** 2026-03-27

**Authors:** Dingxian He, Hongxi Chen, Huahua Zhong, Nana Zhang, Chong Yan, Jie Song, Jianying Xi, Sushan Luo, Chongbo Zhao, Hongyu Zhou

**Affiliations:** 1 Huashan Rare Disease Centre and Department of Neurology, Huashan Hospital, Shanghai Medical College, National Centre for Neurological Disorders, Fudan University, Shanghai, China; 2 Department of Neurology, West China Hospital, Sichuan University, Chengdu, China

**Keywords:** AChR antibody, exacerbation, myasthenia gravis, personalized, prediction

## Abstract

**Background:**

Predicting exacerbation risk in clinically treated patients with myasthenia gravis (MG) is essential for personalised intervention with neurotherapies. This study investigated whether dynamic monitoring of anti-acetylcholine receptor antibody (AChR-ab) levels could predict exacerbation in MG patients following minimal symptom expression (MSE), and developed a validated model for risk stratification to inform treatment decisions.

**Methods:**

We conducted a bicenter cohort study enrolling AChR-ab+ adult MG patients who achieved MSE. The derivation cohort included 339 patients from Huashan Hospital, and the validation cohort comprised 60 patients from West China Hospital. Exacerbation was defined as an increase of ≥ 2 points in the MG Activities of Daily Living score. Independent predictors were identified through univariate and multivariate logistic regression analyses, and a prediction model was subsequently developed and validated using discrimination and calibration metrics.

**Results:**

Longitudinal AChR-ab levels strongly correlated with disease progression, with a median time to exacerbation of 35.1 months post-MSE. Independent predictors included disease duration to MSE achievement, AChR-ab change, thymoma, comorbid immune-related diseases, and history of myasthenic crisis. The model demonstrated excellent discrimination in derivation (AUC = 0.886) and validation cohorts (AUC = 0.829), with good calibration in both datasets.

**Conclusion:**

Dynamic AChR-ab monitoring provides strong predictive value for MG exacerbation, and our validated prediction model offers clinicians a practical tool for personalized risk stratification and therapeutic decision-making in patients achieving disease stability.

## 1 Introduction

Myasthenia gravis (MG) is an antibody-mediated autoimmune disease characterized by fluctuating muscle weakness and fatigability, with approximately 85% of cases involving antibodies against nicotinic acetylcholine receptors (AChR) at the neuromuscular junction (1). The disease imposes a substantial burden on patients and healthcare systems globally. In China, the age- and sex-adjusted incidence of MG is 0.68 per 100,000 person-years, with an admission mortality rate of 14.69‰ (2). Beyond clinical manifestations, patients with MG experience significantly impaired health-related quality of life, considerable economic burden, psychological distress, and increased disability rates (3–7). A primary therapeutic goal in MG management is achieving minimal symptom expression (MSE), which represents optimal disease control and improved quality of life (8). However, disease stability does not equate to cure, and a substantial proportion of patients remain at risk for exacerbation.

The ability to predict exacerbation risk in clinically stable patients has profound therapeutic implications. Despite achieving MSE, over half of MG patients experience exacerbation within five years (9), with approximately 12% developing myasthenic crisis that carries 4-5% in-hospital mortality (10, 11). Currently, treatment tapering decisions are made empirically without reliable predictive tools, resulting in relapse rates approaching 50% during immunosuppressant reduction (4, 12). Identifying patients at high risk for poor prognosis would enable early intervention before clinical deterioration, while low-risk patients could be candidates for cautious treatment tapering. This shift from reactive to proactive management represents a critical unmet need in MG care.

Current prediction models in MG are predominantly designed for acute or transitional disease phases, including short-term outcome prediction, crisis risk assessment, generalization probability, and post-thymectomy prognosis (13–16). These models do not adequately address the unique challenge of predicting exacerbation in clinically stable, post-MSE patients. Traditional monitoring approaches, including clinical assessments, repetitive nerve stimulation tests, and MG-associated scales, are limited by delayed detection and subjective interpretation. Although novel serological markers such as microRNAs, inflammatory cytokines, and complement components have shown promise for disease tracking (17–20), their clinical utility for post-MSE exacerbation prediction remains to be established. Thus, there is an urgent need to identify reliable and readily accessible biomarkers that can accurately predict disease progression and guide therapeutic decisions in this population.

Anti-AChR antibodies (AChR-ab), belonging primarily to the IgG1 and IgG3 subclasses, exert pathogenic effects through complement activation, receptor crosslinking, and direct binding site blockade at the postsynaptic membrane (21–23). While prior studies employing various serological testing approaches have demonstrated that static AChR-ab titers at individual timepoints show poor correlation with clinical severity and limited ability to predict relapse, growing evidence supports the prognostic value of dynamic antibody monitoring (24, 25). Longitudinal studies have demonstrated positive correlations between AChR-ab concentrations and disease severity scores during immunosuppressive treatment (26). Furthermore, perioperative AChR-ab elevations have been associated with postoperative exacerbation in patients with thymoma (27), and higher antibody reduction rates correlate with better clinical outcomes at follow-up (28). These findings suggest that the magnitude of antibody change, rather than absolute levels, may more accurately reflect therapeutic response and predict clinical trajectory.

In this study, we leveraged longitudinal AChR-ab monitoring data from a large MG cohort to: (1) investigate the correlation between the magnitude of AChR-ab level changes (ΔAChR-ab, nmol/L) and the degree of clinical improvement or exacerbation in MG patients; and (2) develop and validate a prediction model that integrates antibody dynamics with clinical variables to enable personalized risk stratification and guide therapeutic decision-making in post-MSE MG patients.

## 2 Materials and methods

### 2.1 Study cohort

**Figure 1:**
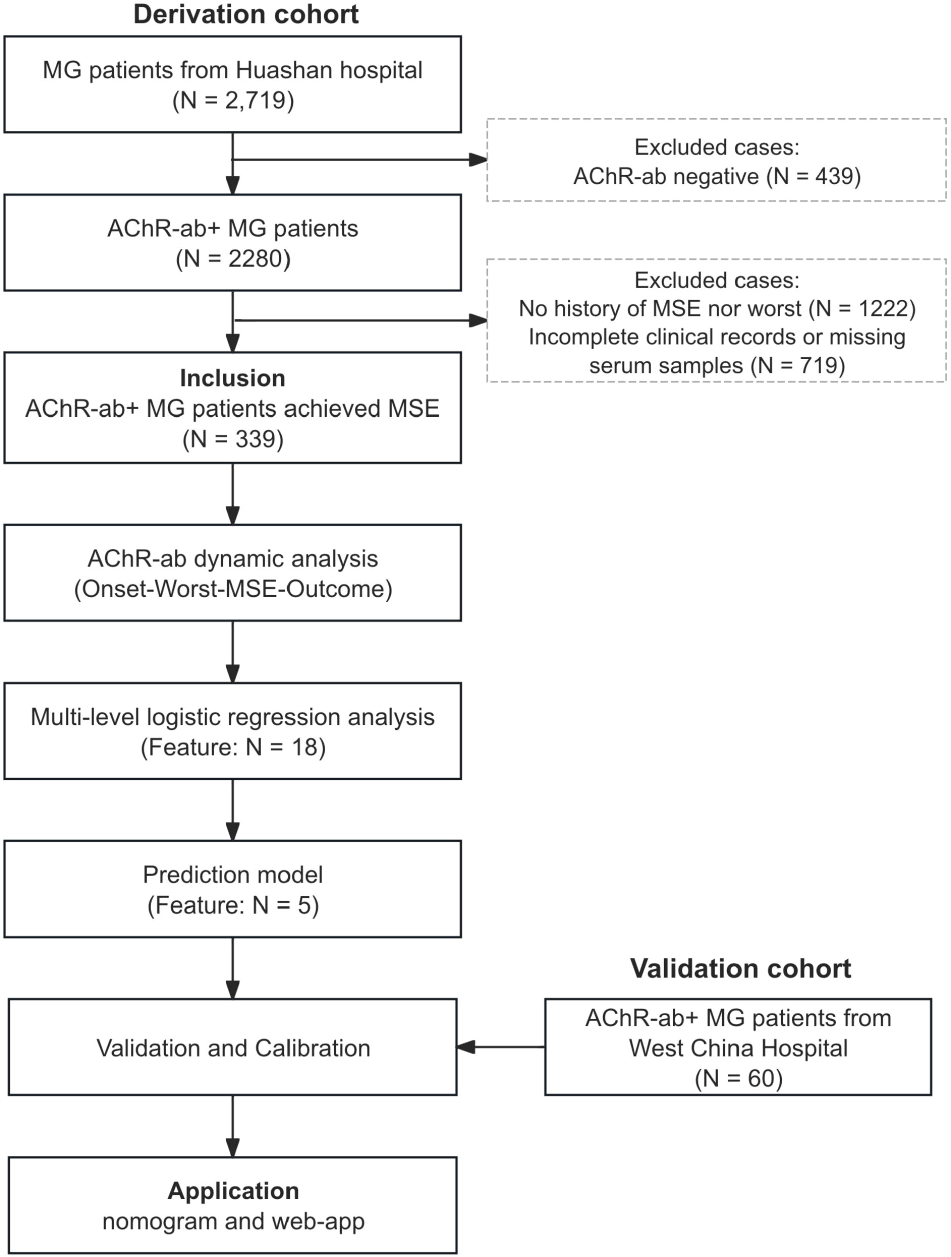
Study flowchart showing patient selection and model development process. The derivation cohort was established from 2,719 MG patients at Huashan Hospital. After excluding AChR-ab negative patients (N = 439), patients without history of MSE or worst stage (N = 1,222), and those with incomplete clinical records or missing serum samples (N = 719), a total of 339 AChR-ab+ MG patients who achieved MSE were included for analysis. Feature selection identified 18 relevant variables, which were further refined to 5 variables for the final prediction model. The model was validated and calibrated using an independent validation cohort of 60 AChR-ab+ MG patients from West China Hospital. The validated model was then applied for clinical prediction purposes. MG, Myasthenia Gravis, AChR-ab, acetylcholine receptor antibody; MSE, minimal symptom expression;.

We conducted a cohort-based study to develop and validate a prediction model for clinical exacerbation risk in MG patients (**Figure 1**). The derivation cohort comprised 339 patients from the Huashan MG Registry (January 1, 2015 through August 31, 2025), a disease-specific database containing 2,719 MG patients. The validation cohort included 60 patients from MG Case Registry System of West China Hospital (January 1, 2015 through August 31, 2025), containing 2059 patients. No individual patient was repeatedly enrolled. MG diagnosis was established according to MGFA 2020 criteria, with study inclusion limited to adult AChR-ab positive (AChR-ab+) patients who had achieved MSE (29). Patients with incomplete clinical records or missing serum samples were excluded. The study was approved by the Ethics Committees of Huashan Hospital (2019-441, 2020–999 and 2022-913) and West China Hospital (2019–536 and 2024-1700). All participants provided written informed consent. The study adhered to the principles of the Declaration of Helsinki.

### 2.2 Follow-up and clinical assessment

**Figure 2:**
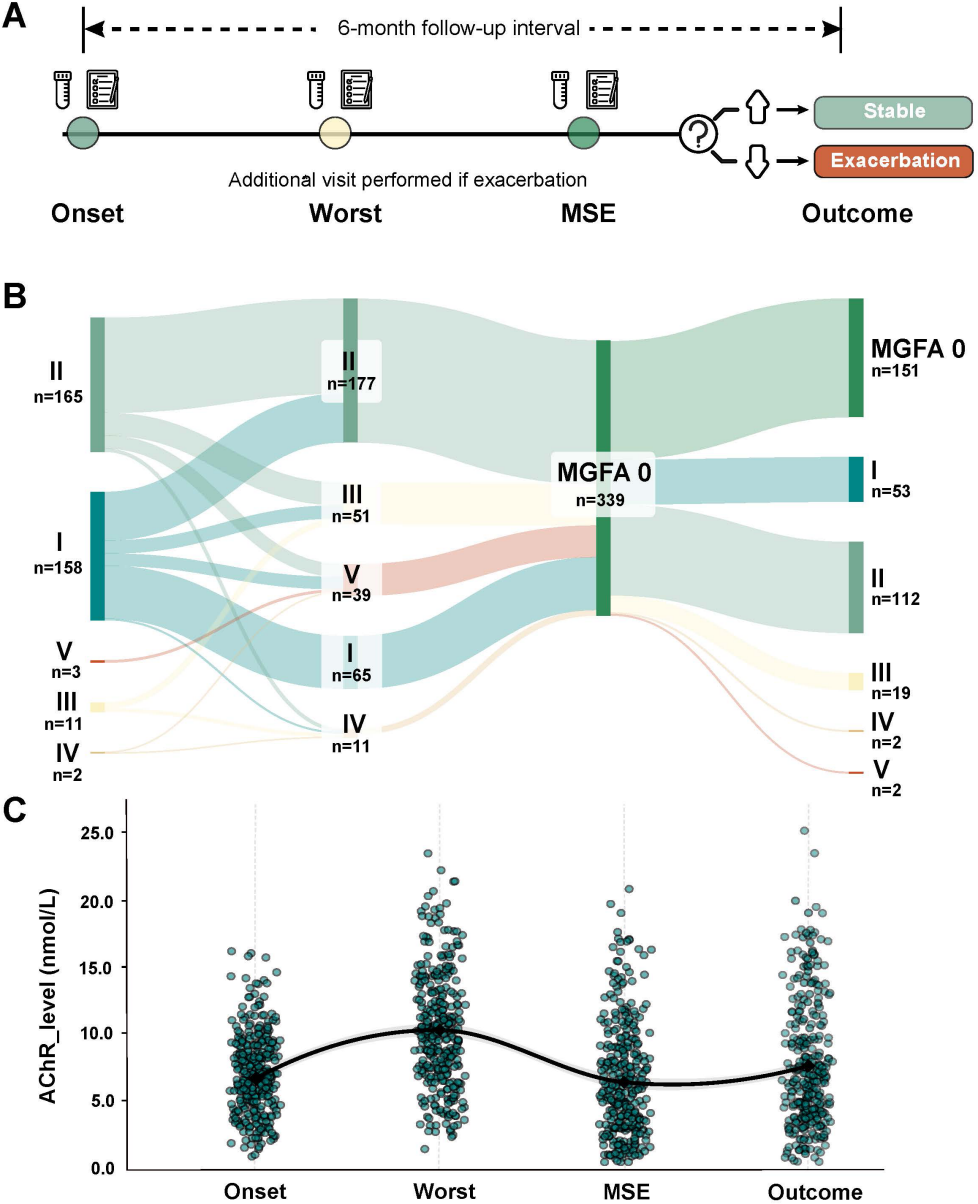
Disease progression and AChR-ab dynamics **(A)** The upper timeline shows the standardized 6-month follow-up protocol with clinical evaluations and serum collection. Additional visit were performed if exacerbation occured. **(B)** The Sankey diagram depicts MGFA classification changes throughout the disease course (n=339), with flow width proportional to patient numbers. **(C)** Longitudinal AChR-ab levels across disease stages. The LOESS regression curve illustrates the temporal trend of AChR-ab levels across four disease stages (Onset, Worst, MSE, Outcome). MSE, minimal symptom expression; MGFA, Myasthenia Gravis Foundation of America; AChR-ab, acetylcholine receptor antibody.

Follow-up assessments were prospectively conducted at standardised 6-month intervals. Additional visits are arranged for patients experiencing symptom exacerbation or clinical deterioration, ensuring comprehensive capture of disease progression events (**Figure 2A**).

We identified four key clinical time-points for each patient: (1) Onset stage, defined as the initial appearance of MG symptoms before any immunosuppressive therapy; (2) Worst stage, defined as the peak severity stage between Onset and MSE stage; (3) MSE stage, defined as achievement of MG-ADL score 0 or 1; and (4) Outcome stage, performed with data censored on August 31, 2025, with patients having mean follow-up of 71.5 ± 64.11 months. Based on MG-ADL score changes from MSE achievement to final assessment, patients were stratified into an exacerbation group (≥2-point increase) and a stable group (<2-point increase). All patients received corticosteroid therapy as part of standard MG management, with a subset additionally receiving non-steroidal immunosuppressive agents (NSISTs). Detailed dosing and tapering information were not systematically recorded.

### 2.3 Clinical data collection

At each visit, clinical features were systematically collected, including demographics (sex, age at screening, onset age), disease characteristics (thymoma status, thymectomy history, clinical subtypes), treatment regimens, and standardized assessment scores including MG-ADL, Quantitative Myasthenia Gravis Score (QMG), Myasthenia Gravis Composite Score (MGC), and Myasthenia Gravis Quality of Life 15-item scale (MG-QOL-15).

### 2.4 Antibody testing methodology

Peripheral blood serum samples were obtained at each visit and were stored at -80 °C refrigerator. Serum AChR-ab levels were quantified using radioimmunoprecipitation assay (RIPA), the gold standard method for AChR antibody detection. This assay utilizes radiolabeled human AChR as antigen, with antibody-antigen complexes precipitated and quantified by gamma counting. All analyses were conducted at Kindstar Global laboratory with results expressed as nmol/L (30).

For analysis, antibody data were specifically selected from the four key time-points: (1) Onset: baseline antibody levels before treatment; (2) Worst: antibody levels at peak disease severity before MSE; (3) MSE: antibody levels upon achieving minimal symptom expression; and (4) Outcome: antibody levels at the time of first exacerbation following MSE, or at the final follow-up visit for stable patients.

### 2.5 Definitions

For this cohort analysis, clinical definitions were established as follows. MG exacerbation was defined as the reappearance or exacerbation of muscle weakness symptoms lasting >24 hours and MG-ADL≥2-point increase, while stable disease was defined as sustained MSE throughout follow-up (31). MSE was defined as MG-ADL score 0–1 or Myasthenia Gravis Foundation of America (MGFA) class 0 (asymptomatic status) (32, 33). Myasthenic crisis (MC) was defined as rapid clinical deterioration requiring non-invasive or invasive ventilation, while impending MC was defined as rapid clinical exacerbation that could progress to crisis within days to weeks, as determined by the treating physician (34, 35). Comorbid immune-related diseases were classified according to ICD-10 codes, including autoimmune conditions (autoimmune thyroid diseases, rheumatoid arthritis, systemic lupus erythematosus, inflammatory bowel disease, and other autoimmune disorders) and chronic immune-relevant infections (hepatitis B), the latter of which was included based on evidence linking chronic HBV infection to persistent immune activation and potential modulation of autoimmune disease course. Thymoma was analyzed as a separate variable given its distinct pathophysiological relationship with MG, with diagnosis requiring histological verification (36). Based on disease trajectory after achieving MSE, patients were categorized into two groups: stable group (maintained MSE without exacerbation) versus exacerbation group (experienced disease worsening as defined above).

### 2.6 Model derivation and validation

Nomogram development followed a systematic approach adhering to TRIPOD guidelines: Initially, univariate logistic regression identified candidate variables with liberal significance (p < 0.1) to minimize exclusion of potentially relevant predictors. Considering the events-per-variable ratio, these candidates underwent backward stepwise multivariate logistic regression with stringent criteria (p < 0.05) to determine independent predictors for nomogram construction.

Model performance was comprehensively evaluated through discrimination and calibration metrics. Discrimination ability was quantified via AUC-ROC analysis with 95% confidence intervals across both cohorts. Calibration quality was examined using Hosmer-Lemeshow goodness-of-fit testing and graphical calibration assessment. Model validation employed by external validation computing discriminative performance and calibration metrics in the independent validation cohort to evaluate model generalizability. The interactive calculator is publicly available online.

### 2.7 Statistical analysis

Statistical analyses were conducted using RStudio (version 4.4.2, Posit, USA) or GraphPad Prism (version 10.4.0, GraphPad Software Inc., USA). Continuous variables were assessed for normality using the Shapiro–Wilk test. Non‐normally distributed data were presented as the median (interquartile range, IQR) and compared using Mann-Whitney U tests or Kruskal-Wallis tests. Normally distributed data were reported as means ± standard deviations (SDs) and compared using T-tests or analysis of variance (ANOVA). Categorical variables were reported as percentages (%) and were compared using chi-square tests or Fisher’s exact tests, depending on sample size.

Correlation analysis was performed to examine the relationship between the magnitude of ΔAChR-ab and the degree of clinical improvement or exacerbation in MG patients, using Pearson or Spearman correlation coefficients based on data distribution. Time-to-event analysis for disease progression from the MSE stage to exacerbation was conducted using Kaplan-Meier survival curves with log-rank tests. The 35.1-month time horizon for binary endpoint modeling was derived from the observed median time to exacerbation in the Kaplan-Meier analysis and should be considered exploratory rather than pre-specified. Statistical significance was defined as a two-sided p < 0.05.

## 3 Results

### 3.1 Demographic features and clinical manifestations

A total of 339 MG patients fulfilled the inclusion criteria, with their baseline demographic and clinical characteristics summarized in **Table 1**. The cohort comprised 63.1% female (214/339) and 36.9% male (125/339) patients. The median age at disease onset was 41.0 years (IQR: 28.0-54.0), and patients achieved MSE stage at a median age of 44.0 years (IQR: 31.0-58.0).

**Table 1:** Demographic features and clinical manifestation.

Variables	Derivation cohort (n = 339)	Validation cohort (n = 60)	P value
Sex, n (%)			0.127
Female	214 (63.1)	44 (73.3)	
Male	125 (36.9)	16 (26.7)	
Age at onset stage (years), Median (Q1, Q3)	41.0 (28.0, 54.0)	40.0 (29.5, 50.5)	0.445
Age at MSE stage (years), Median (Q1, Q3)	44.0 (31.0, 58.0)	42.0 (31.5, 52.0)	0.400
Duration (onset to MSE stage) (months), mean ± SD	45.8 ± 61.05	45.8 ± 64.91	0.997
AChR-Ab level (nmol/L) at onset, Median (Q1, Q3)	6.6 (4.6, 8.7)	6.2 (4.0, 8.4)	0.548
AChR-Ab level (nmol/L) at worst, Median (Q1, Q3)	10.1 (7.4, 13.1)	9.6 (5.5, 14.0)	0.543
AChR-Ab level (nmol/L) at MSE, Median (Q1, Q3)	5.9 (3.2, 9.1)	5.6 (3.5, 9.2)	0.561
Thymoma, n (%)			0.682
Yes	104 (30.7)	20 (33.3)	
None	235 (69.3)	40 (66.7)	
Thymectomy, n (%)			0.695
Yes	121 (35.7)	23 (38.3)	
None	218 (64.3)	37 (61.7)	
Comorbid IRDs, n (%)			0.401
Yes	122 (36.0)	25 (41.7)	
None	217 (64.0)	35 (58.3)	
Onset MG subtype, n (%)			0.479
Ocular MG	158 (46.7)	25 (41.7)	
Generalized MG	181 (53.4)	35 (58.3)	
MG-ADL at MSE, Median (Q1, Q3)	0.0 (0.0, 0.0)	0.0 (0.0, 0.0)	0.914
QMG at MSE, Median (Q1, Q3)	4.0 (2.0, 6.0)	4.0 (1.0, 6.0)	0.712
MGC at MSE, Median (Q1, Q3)	0.0 (0.0, 1.0)	0.0 (0.0, 1.5)	0.325
MG-Qol-15 at MSE, Median (Q1, Q3)	1.0 (0.0, 6.0)	1.0 (0.0, 10.0)	0.573
Crisis1, n (%)			0.689
Yes	46 (13.6)	7 (11.7)	
None	293 (86.4)	53 (88.3)	
NSISTs administration, n (%)			0.697
Yes	190 (56.0)	32 (53.3)	
None	149 (44.0)	28 (46.7)	

^1^ Crisis:History of MC (requiring ventilation) or impending MC (rapid exacerbation potentially progressing to crisis).

AChR-ab, acetylcholine receptor antibody; NSISTs, non-steroid immunosuppressant therapy; MG, myasthenia gravis; MG-ADL, Myasthenia Gravis Activities of Daily Living scale; MGC, Myasthenia Gravis Composite scale; MG-QoL-15, Myasthenia Gravis Quality of Life 15-item scale; MSE, minimal symptom expression; Q1, first quartile; Q3, third quartile; QMG, Quantitative Myasthenia Gravis score; MC, myasthenic crisis.

At onset, 53.4% of patients (181/339) exhibited generalized MG symptoms, while 46.7% of patients (158/339) presented with ocular symptoms only. The median AChR-ab level at onset stage was 6.6 nmol/L (IQR: 4.6-8.7). The Sankey diagram illustrates the dynamic disease progression from onset through worst stage to MSE (**Figure 2B**). Patients progressed from onset to MSE stage after a mean duration of 45.8 ± 61.05 months. By the MSE stage, all patients had achieved asymptomatic status. During this progression, AChR-ab levels peaked at the worst stage with a median of 10.1 nmol/L (IQR: 7.4-13.1), then decreased to 5.9 nmol/L (IQR: 3.2-9.1) at MSE stage.

Thymoma was identified in 30.7% of patients (104/339), with WHO type B2 being the most common subtype (18.6%, 63/339). Thymectomy was performed in 35.7% of patients (121/339). Comorbid immune-related diseases were present in 36.0% of patients (122/339). During the disease course prior to achieving MSE, 13.6% of patients (46/339) experienced myasthenic crisis or impending crisis, and 56.0% of patients (190/339) required non-steroidal immunosuppressive therapy in addition to steroid treatment. Based on clinical changes from MSE to follow-up outcome (duration, 25.64 ± 18.63 month), patients were categorized into Stable group (44.5%, 151/339) and Exacerbation group (55.5%, 188/339).

### 3.2 Longitudinal changes in AChR-ab levels correlate with disease progression in MG

**Figure 3:**
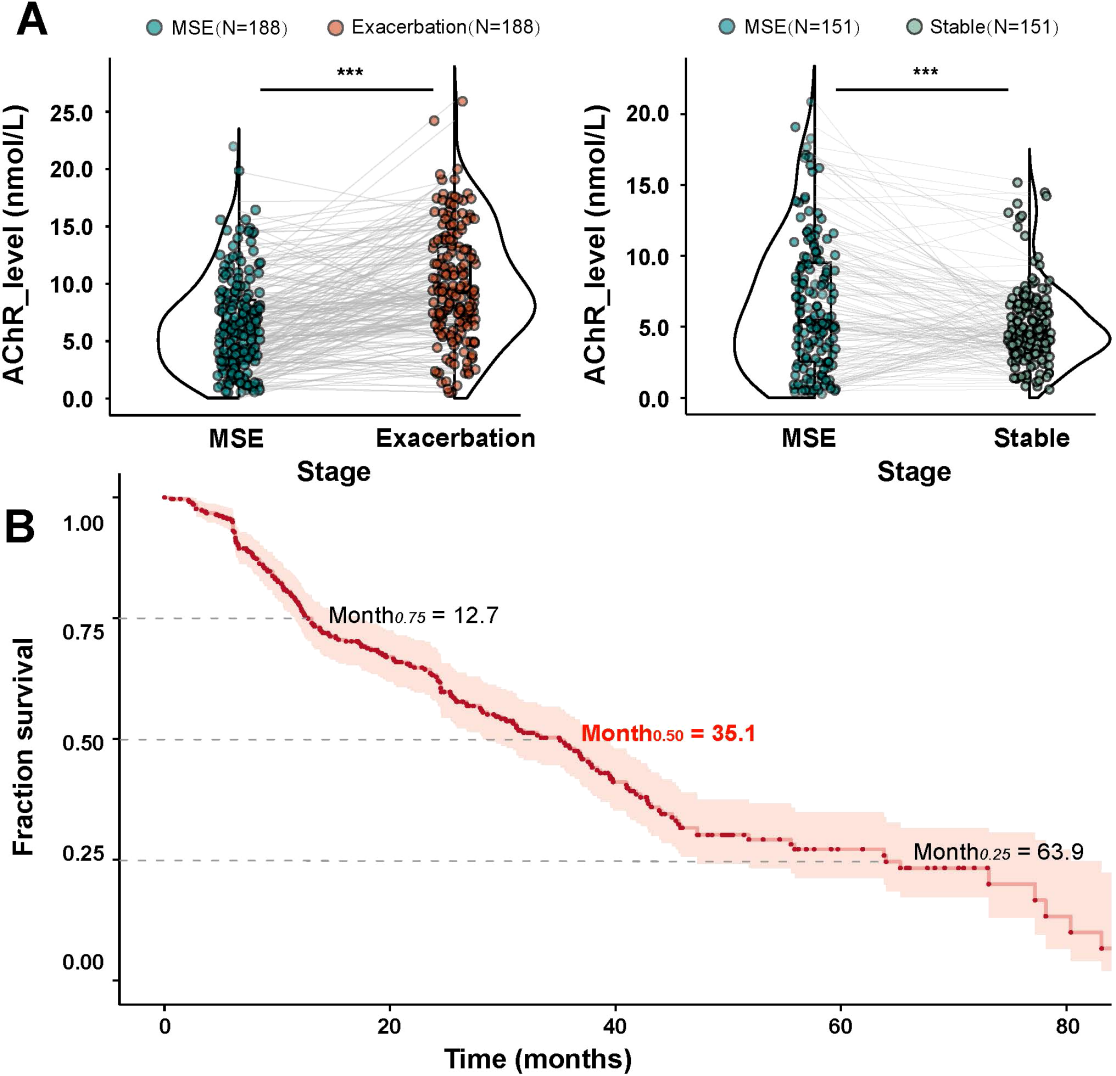
AChR-ab Level Changes and Exacerbation Risk after MSE **(A)** The violin plots compare AChR-ab levels between MSE vs. exacerbation (left, n=188) and MSE vs. stable (right, n=151). **(B)** Kaplan-Meier analysis for time to exacerbation. Median time: 35.1 months (25th-75th percentile: 12.7-63.9 months). MG, Myasthenia Gravis, AChR-ab, Acetylcholine receptor antibody; MSE, minimal symptom expression;.

LOESS regression analysis revealed a distinct disease activity-related pattern of AChR-ab level variations across the disease course (**Figure 2C**). The smoothed curve revealed a progression from intermediate antibody concentrations at MG onset to peak levels during the worst disease stage, followed by lower concentrations during the MSE phase. Using MSE as the reference point (**Figure 3A**), patients demonstrated significantly elevated AChR-ab levels during exacerbations (p<0.001) and reduced levels during stable periods (p<0.001) compared to their individual MSE values.

**Figure 4:**
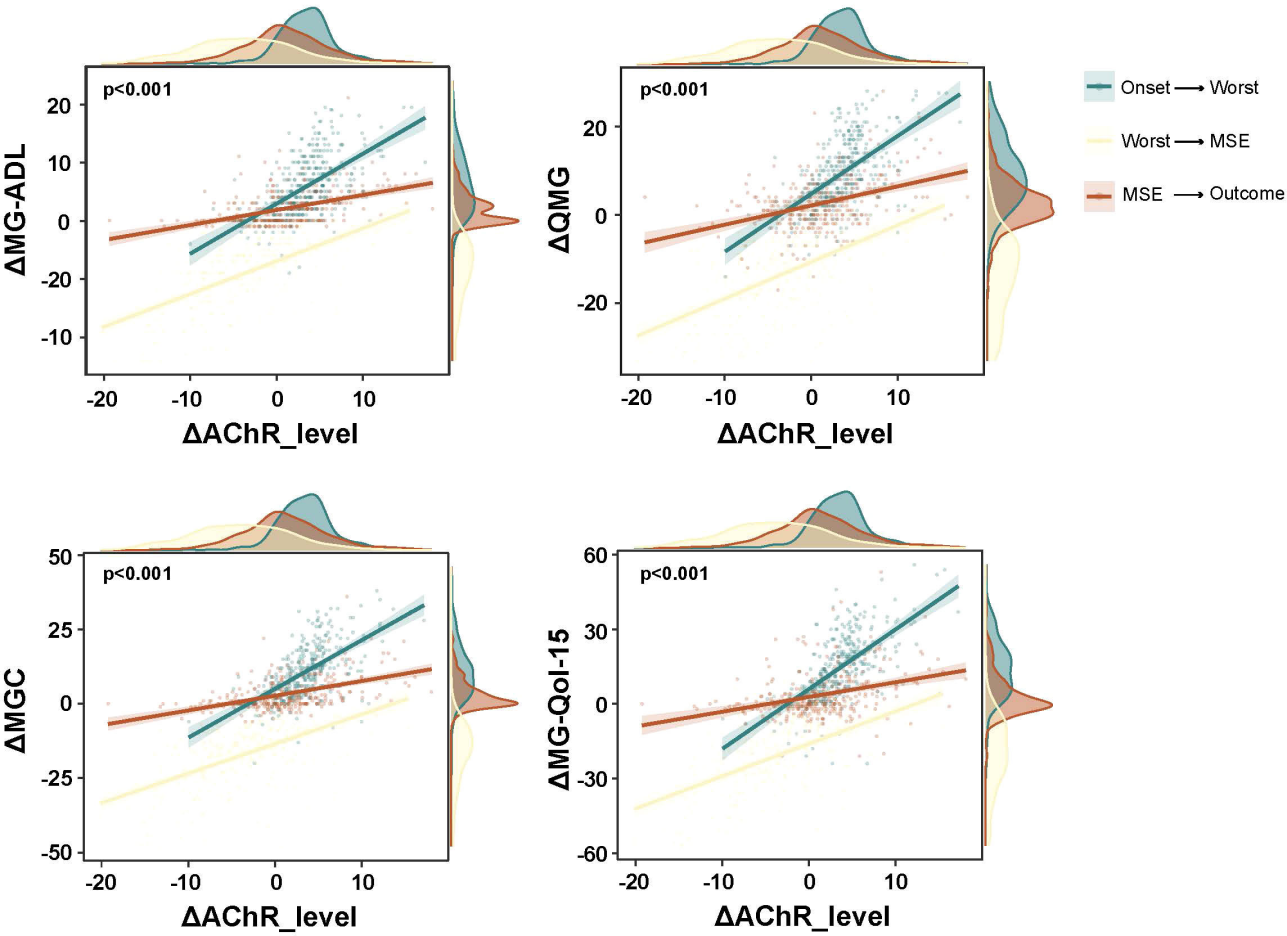
Correlation between AChR antibody levels and clinical score changes. Scatter plots show the relationship between changes in AChR-ab levels (x-axis) and changes in four clinical assessment scores (y-axis): MG-ADL, QMG, MGC, and MG-QOL-15. Data points represent individual patients across three disease transition phases: Onset→Worst, Worst→MSE, and MSE→Outcome. Regression lines with 95% confidence intervals are shown. All correlations were statistically significant (p<0.001). AChR, acetylcholine receptor; MSE, minimal symptom expression; MG-ADL, Myasthenia Gravis Activities of Daily Living; QMG, Quantitative Myasthenia Gravis; MGC, Myasthenia Gravis Composite; MG-Qol-15, Myasthenia Gravis Quality of Life-15.

After achieving MSE, the Kaplan-Meier survival analysis identified AChR-ab levels as a significant predictor of disease exacerbation (**Figure 3B**). The median time to exacerbation following MSE was 35.1 months, with 25% and 75% of patients experiencing exacerbation at 12.7 and 63.9 months, respectively. Furthermore, strong correlations were observed between AChR-ab level fluctuations and validated clinical outcome measures across all three disease phases (MG-ADL: p<0.001; QMG: p<0.001; MGC: p<0.001; MG-Qol-15r: p<0.001) (**Figure 4**). These findings confirm that longitudinal AChR-ab levels may serve as reliable biomarkers for tracking disease progression and predicting exacerbation risk in MG.

### 3.3 Derivation and validation of the nomogram

Kaplan-Meier analysis revealed that half of the patients experienced symptom exacerbation at 35.1 months following MSE achievement (**Figure 3B**), which we established as the optimal time horizon for predictive modeling. Univariate analysis identified several variables associated with exacerbation risk (**Table 2**). Variables with p<0.1 in univariate analysis were subsequently included in the multivariable regression model. Significant risk factors for exacerbation included shorter disease onset to MSE duration (Exacerbation vs. Stable, 36.3 ± 42.4 vs. 50.9 ± 68.1 months; p=0.023), smaller AChR-ab reduction (Exacerbation vs. Stable, ΔAChR-ab: 2.60 ± 2.88 vs. 3.59 ± 3.73 nmol/L; p=0.041), thymoma (p<0.001), thymectomy (p=0.004), comorbid immune-related diseases (IRDs) (p=0.047), and history of crisis/impending crisis (p=0.056). Given the collinearity between thymoma and thymectomy variables (VIF = 14.1), we retained thymoma as the primary predictor in our final model.

**Table 2:** Baseline characteristics of patients stratified by exacerbation status within 35.1 months post-MSE.

Variables	Exacerbation (n=144)	Stable (n=195)	P value
Sex, n (%)			0.927
Female	90 (62.5)	124 (63.6)	
Male	54 (37.5)	71 (36.4)	
Age at onset stage (years), Median (Q1, Q3)	42.5 (28.0, 55.8)	41.0 (28.0, 54.0)	0.132
Age at MSE stage (years), Median (Q1, Q3)	46.5 (31.0, 59.0)	41.0 (28.0, 54.0)	0.235
Duration (onset to MSE stage) (months), mean ± SD	36.3 ± 42.4	50.9 ± 68.1	0.023
ΔAChR-ab level (Onset to MSE) (nmol/L), mean ± SD	2.60 ± 2.88	3.59 ± 3.73	0.041
Thymoma, n (%)			<0.001
Yes	60 (41.7)	44 (22.6)	
None	84 (58.3)	151 (77.4)	
Thymectomy, n (%)			0.004
Yes	64 (44.4)	57 (29.2)	
None	80 (55.6)	138 (70.8)	
Comorbid immune-related diseases, n (%)			0.047
Yes	61 (42.4)	61 (31.3)	
None	83 (57.6)	134 (68.7)	
Onset MG subtype, n (%)			0.599
Ocular MG	70 (48.6)	88 (45.1)	
Generalized MG	74 (51.4)	107 (54.9)	
MG-ADL at MSE stage, Median (Q1, Q3)	0 (0, 0)	0 (0, 0)	0.661
QMG at MSE stage, Median (Q1, Q3)	4 (2, 7)	3 (1, 6)	0.779
MGC at MSE stage, Median (Q1, Q3)	0 (0, 1)	0 (0, 1)	0.404
MG-Qol-15 at MSE stage, Median (Q1, Q3)	3 (0, 6)	3 (1, 5)	0.622
Crisis1, n (%)			0.056
Yes	26 (18.1)	20 (10.3)	
None	118 (81.9)	175 (89.7)	
NSISTs administration, n (%)			0.133
Yes	88 (61.1)	102 (52.3)	
None	56 (38.9)	93 (47.7)	

^1^ Crisis:History of MC (requiring ventilation) or impending MC (rapid exacerbation potentially progressing to crisis).

AChR-ab, acetylcholine receptor antibody; NSISTs, non-steroid immunosuppressant therapy; MG, myasthenia gravis; MG-ADL, Myasthenia Gravis Activities of Daily Living scale; MGC, Myasthenia Gravis Composite scale; MG-QoL-15, Myasthenia Gravis Quality of Life 15-item scale; MSE, minimal symptom expression; Q1, first quartile; Q3, third quartile; QMG, Quantitative Myasthenia Gravis score; SD, standard deviation; WHO, World Health Organization.

**Figure 5:**
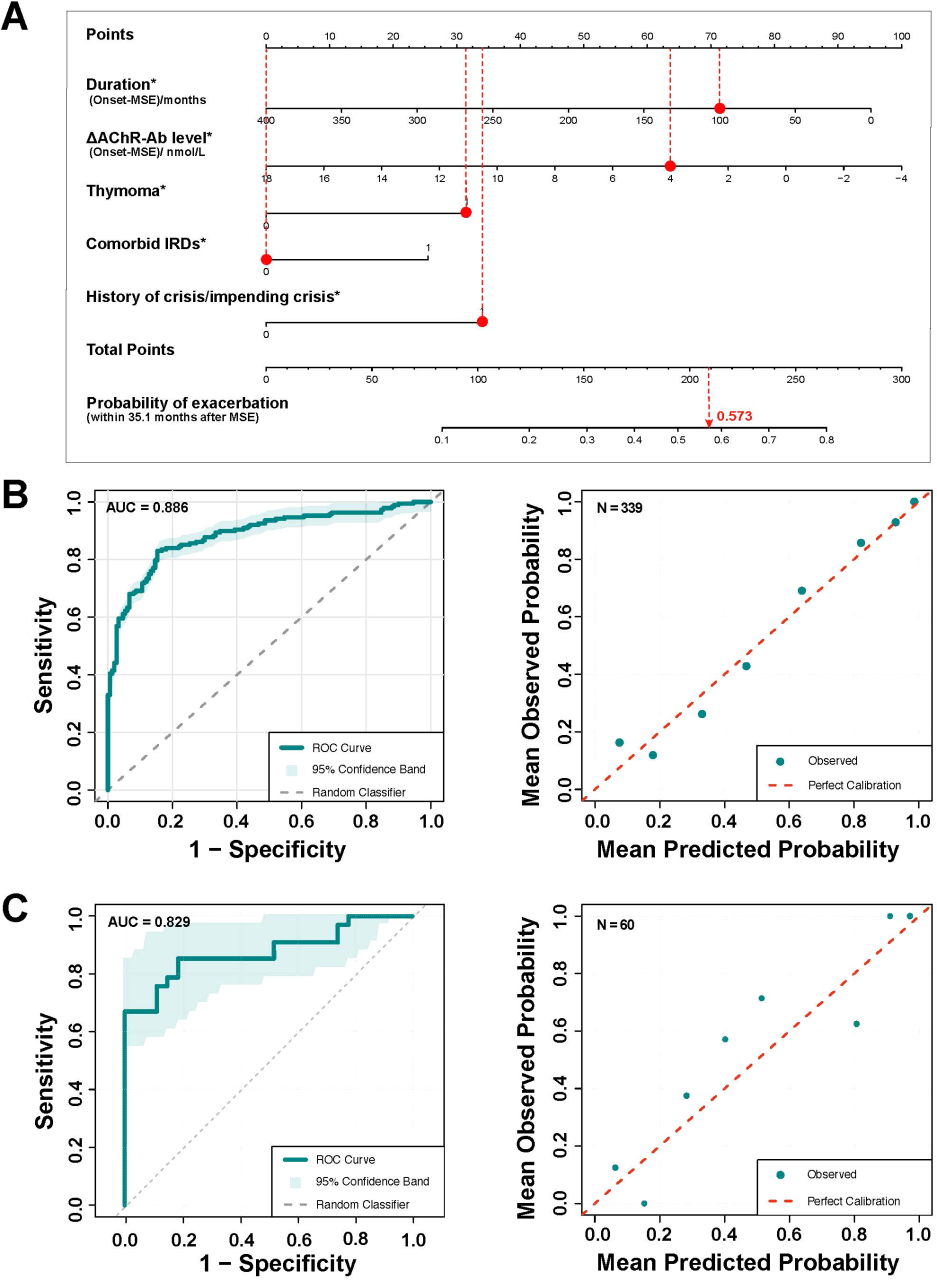
Development and validation of the prediction model for exacerbation risk. Nomogram to estimate the exacerbation risk in AChR-ab+ MG patients. **(B)** The ROC-AUC and model calibration of derivation cohort: AUC = 0.886, 95% CI: 0.852–0.921. **(C)** The ROC-AUC and model calibration of validation cohort: AUC = 0.829, 95% CI: 0.721–0.936. MSE: minimal symptom expression, ΔAChR-ab: change of acetylcholine receptors antibody from onset to MSE, IRD: immune-related disease, AUC: Area under the receiver operating characteristic curve.

In the final multivariable logistic regression model, we identified independent predictors of symptom exacerbation within 35.1 months post-MSE and established the following prediction equation: logit(P) = -0.3779 - 0.0047 × Duration (p=0.039) - 0.0914 × ΔAChR-ab (p=0.011) + 0.6027 × Thymoma (p=0.015) + 0.4941 × Comorbid IRDs (p=0.040) + 0.9029 × History of crisis/impending crisis (p=0.044) (**Figure 5A**). Subsequently, the nomogram demonstrated high discriminatory power (AUC = 0.886, 95% CI: 0.852–0.921) and good calibration (Hosmer-Lemeshow goodness-of-fit test, χ² = 3.869, p = 0.868) in the derivation cohort (**Figure 5B**). External validation confirmed robust model performance (**Table 1**), maintaining good discrimination (AUC = 0.829, 95% CI: 0.721–0.936) and adequate calibration (Hosmer-Lemeshow test: χ² = 6.261, p = 0.395), thereby confirming the nomogram’s clinical utility (**Figure 5C**). To enhance clinical accessibility, we developed an interactive online calculator enabling real-time prediction of exacerbation probability within 35.1 months post-MSE at the point of visit. The prediction model is available as a web-app at https://demo.huashanmuscle.com/exacerbation_prediction_nomogram.html.

## 4 Discussion

Identifying patients at risk for exacerbation is essential for guiding therapeutic decisions in MG, particularly in determining the appropriate intensity and duration of immunosuppressive therapy. This multicenter study of 399 MG patients demonstrates that dynamic AChR-ab monitoring represents a paradigm shift from traditional static assessments to longitudinal biomarker tracking. While previous studies have primarily focused on static AChR-ab levels at diagnosis or single time points (37, 38), our dynamic approach enabled development of a five-factor (a shorter disease duration, ΔAChR-ab, thymoma, comorbid IRDs and history of crisis/impending crisis) nomogram specifically predicting exacerbation probability within 35.1 months post-MSE, achieving exceptional discriminatory performance (AUC = 0.886) with robust external validation (AUC = 0.829). The resulting interactive online calculator provides clinicians with a validated tool for personalized risk assessment of disease exacerbation, filling the gap in using readily available serum antibodies for predictive modeling.

LOESS regression analysis revealed a clear dynamic relationship between AChR-ab levels and disease activity, demonstrating that antibody concentrations peak during exacerbations and reach nadir levels during clinical remission. This pattern supports the concept that antibody kinetics, rather than static absolute values, provide superior prognostic information. Supporting evidence comes from multiple studies: Kojima et al. demonstrated a strong correlation between declines in RR-AChR-ab rates and improvements in MG-ADL scores, while Luo et al. observed that decreases in AChR-ab levels coincided with reductions in QMGS and ADL scores following standardized treatment initiation in MG patients (28, 39). Notably, our ΔAChR-ab parameter represents the antibody level change from disease onset to MSE achievement, effectively capturing the immunological response to therapeutic intervention and serving as a composite biomarker of treatment efficacy. The pathophysiological rationale for ΔAChR-ab as a biomarker stems from the complex interplay between antibody production, clearance, and target engagement at the NMJ. AChR-abs mediate pathogenic effects via three main mechanisms: (1) complement-mediated focal endplate lysis, (2) crosslinking-induced receptor internalization and degradation, and (3) direct acetylcholine binding site blockade (40). Dynamic biomarker monitoring has proven effective across multiple autoimmune conditions: In rheumatoid arthritis, serial anti-CCP antibody monitoring correlates with treatment response, while in systemic lupus erythematosus, anti-dsDNA antibody fluctuations serve as validated predictors of disease flares (41–43). Similarly, longitudinal neurofilament light chain monitoring in multiple sclerosis has emerged as a powerful tool for assessing treatment efficacy (44). These precedents validate the broader applicability of dynamic biomarker strategies in autoimmune disease management and support our MG-specific approach.

It is important to acknowledge that in the context of MG, clinical improvement and antibody reduction are both largely consequences of immunotherapies, while the two phenomena are inherently difficult to disentangle. As Kuks et al. observed, fluctuations in AChR-ab levels tend to occur simultaneously with clinical changes under immunosuppressive therapies, reflecting parallel rather than independent processes (45). In our cohort, all patients received corticosteroid as part of standard care, with 56.0% additionally receiving adjunct non-steroidal immunosuppressive agents. Given this uniform treatment background, the relevant question is not whether immunotherapy drives antibody reduction, but why the magnitude of that reduction substantially varies among individuals receiving comparable treatment. In a recent analysis of MGTX trial data, AChR-specific IgG titers declined significantly in patients achieving clinically meaningful improvement; in contrast, they remained stable in non-responders (46). Similarly, the magnitude of post-thymectomy antibody reduction has been shown to significantly correlate with achievement of complete stable remission (p=0.002) (47). These findings suggest that under comparable treatment conditions, the degree of antibody reduction reflects the depth of individual immunological responsiveness rather than serving as a mere surrogate of treatment exposure.

Beyond ΔAChR-ab, the remaining predictive factors exhibit two distinct correlation patterns with exacerbation risk. Early-stage vulnerability emerged as a key pattern, where shorter disease duration correlated with higher exacerbation risk. This aligns with established clinical observations that myasthenic crises frequently occur within the first 2–3 years after disease onset, reflecting immune instability and volatile autoimmune responses characteristic of early-phase disease rather than cumulative pathological burden (48). The early-stage pattern contrasts sharply with factors showing positive correlation with risk, including thymoma, previous crisis episodes, and comorbid IRDs, which represent established markers of persistent immune dysregulation. Specifically, thymoma-associated disease involves continuous production of autoreactive T cells by aberrant thymic epithelial cells, creating a persistent source of immune dysregulation even after thymectomy (49). Meanwhile, previous crisis/impending crisis history indicates chronic immune hyperactivation, as evidenced by persistent pro-inflammatory CD4+ T cell signatures, particularly elevated Th1 and Th17 subsets (50). Furthermore, comorbid IRDs reflect shared pathogenic pathways involving dysregulated B and T cell activation, often driven by common genetic susceptibility and epigenetic modifications (51). Collectively, these findings support a dual-pathway model of MG exacerbation risk: early-phase immune instability versus chronic immune dysfunction in severe disease phenotypes.

Our study had several limitations: (1) The small cohort size limits external validity, necessitating larger multi-center studies for validation. (2) Detailed immunotherapy dosing and tapering data were not systematically recorded, and variability in treatment intensity remains a residual confounder that may have affected antibody dynamics and clinical outcomes. (3) Different AChR-ab subtypes and subunit-specific antibodies were not differentiated in our study, which may have affected the precision of our predictive model, as these subtypes exhibit distinct pathogenic mechanisms and clinical significance. (4) The mechanistic understanding of antibody-outcome relationships remains incomplete, and optimal antibody reduction thresholds require further prospective investigation. (5) The 35.1-month prediction horizon was data-driven, which may have influenced apparent model performance. (6) The ΔAChR-ab variable requires onset-stage antibody data, which may not be available for referred patients or those tested at different laboratories, limiting the model’s generalizability beyond settings with complete longitudinal records. Future studies with larger sample sizes and more refined antibody subgrouping are needed to validate our findings and develop more precise predictive models for clinical application.

## 5 Conclusion

Dynamic AChR-ab monitoring demonstrated strong predictive value for disease exacerbation in MG patients achieving MSE. We developed and validated a prediction model incorporating clinical characteristics and readily available serological markers that accurately predicts exacerbation risk, providing clinicians with a practical tool for personalized risk stratification and enhanced therapeutic decision-making in MG management.

## Data Availability

The original contributions presented in the study are included in the article/supplementary material. Further inquiries can be directed to the corresponding authors.
